# Lipidomic analysis of facial skin surface lipid reveals the causes of pregnancy-related skin barrier weakness

**DOI:** 10.1038/s41598-021-82624-3

**Published:** 2021-02-05

**Authors:** Manli Yang, Mingyue Zhou, Yuan Li, Hong Huang, Yan Jia

**Affiliations:** 1 Beijing Key Laboratory of Plant Resources Research and Development, College of Chemistry and Materials Engineering, Beijing Technology and Business University, Beijing, 100048 China; 2 Key Laboratory of Cosmetic of China National Light Industry, College of Chemistry and Materials Engineering, Beijing Technology and Business University, Beijing, 100048 China; 3 Chongqing Key Laboratory of Translational Research for Cancer Metastasis and Individualized Treatment, Chongqing University Cancer Hospital, Chongqing, China; 4 Department of Obstetrics, Beijing Obstetrics and Gynecology Hospital, Capital Medical University, Beijing, China; 5 Department of Gynecology, Beijing Obstetrics and Gynecology Hospital, Capital Medical University, Beijing, China

**Keywords:** Biological techniques, Biomarkers, Medical research

## Abstract

Self-reported skin discomfort is a common problem during pregnancy, but it is not clear whether skin barrier function is altered in the process. Few studies have described the skin barrier function during pregnancy. In this work, we used highly sensitive and high-resolution ultra performance liquid chromatography-quadrupole time-of-flight mass spectrometry (UPLC-QTOF-MS) to distinguish skin surface lipid (SSL) combined with multivariate analysis of lipids and metabolic changes to determine the relationship between SSL changes and skin physiology during pregnancy in order to better understand the skin condition of pregnant women. The results showed a significant reduction in the total lipid content in pregnant women. A total of 2270 lipids were detected, and the relative abundances of fatty acyls and glycerolipids were significantly reduced, while glycerophospholipids (GPs), sphingolipids, and saccharolipids was significantly increased in the pregnancy group. Multivariate data analysis indicated that 23 entities constituted the most important individual species responsible for the discrimination and phosphatidylcholine was the most abundant lipid in pregnancy group. In addition, compared to SSL profile of control group, it was observed that the average chain length of ceramides and fatty acids both decreased in SSL profile of pregnancy group. The main and most commonly affected pathway was that of GP pathways. These findings indicate that skin lipids are significantly altered in mid-pregnancy compared to the control group. Changes in ostrogen during pregnancy also make the skin more susceptible to inflammatory factors and lead to more fragile and susceptible skin, weakening the skin barrier along with the lipid alterations.

## Introduction

Pregnancy is a period closely related to various endocrines, metabolic, immune, and vascular changes in women’s physiological and pathological skin changes^1^. More than 90% of pregnant women experience physiological changes in skin vasculature, pigmentation, appendages, and degeneration^2^. Pre-existing skin diseases (e.g. psoriasis, and atopic dermatitis [AD]), may worsen or improve during pregnancy, and pregnancy-specific skin diseases, such as intrahepatic cholestasis and pruritic urticarial papules, may occur^3^. Skin diseases during pregnancy have received extensive attention and research^4,5^ and often occur in close association with, changes in skin condition^6^. Therefore, we speculated that the state of the skin changes and is prone to some skin diseases during pregnancy, but at the molecular level, the specific changes that take place remain unknown.

The state of the skin is closely related to the strength of the skin barrier and is also altered by changes in skin metabolism and microflora. Skin lipids maintain the skin barrier by creating a physicochemical barrier, transmitting a complex network of signals from the epidermis and altering the composition of the microbial skin flora^7–9^. In particular, healthy skin relies on a characteristic lipid profile to form a barrier^7,10,11^. Changes in the composition and content of skin lipids will affect the function of the skin barrier^12,13^. Studies have shown that defective skin barrier function is not simply a secondary consequence but rather a key factor in various skin diseases^9,14,15^. When the barrier function of the skin is impaired internally or externally, it manifests as irritation, inflammation, or other skin disorders^6^. Nevertheless, to the best of our knowledge, there are no studies comparing facial skin barrier function in non-pregnant normal women versus pregnant women. In particular, changes in the lipid profile of women’s facial skin during the second trimester of pregnancy have not been reported.

Skin lipidomics involves the qualitative and quantitative analyses of different species and active lipids, which provide support for studying the role of lipids in skin health and disease^7,10,16^. In our previous studies, a powerful analytical technique called ultra-performance liquid chromatography-quadrupole time of flight-mass spectrometry (UPLC-QTOF-MS), was used to investigate skin surface lipid (SSL) variations under different conditions^9,17–19^. The results showed that there were significant differences in SSL composition between different skin states. Based on this, UPLC-QTOF-MS was utilised to unveil the differences in facial SSL between pregnant and non-pregnant control subjects. We aimed to provide a theoretical basis for the development of skin management and related skin care products for pregnant women. The results showed that the average fatty acid (FA) chain length on the skin surface of women in the second trimester was significantly shorter (*p* < 0.05), and the relative phosphatidylcholine (PC) content change was the most significant among the lipid components.

## Materials and methods

### Chemicals and reagents

Distilled water, formic acid, isopropyl alcohol, methanol, acetonitrile, and ammonium formate of LC–MS grade were originate from Thermo Fisher Scientific (Waltham, MA, USA). The SSL-adsorbent Sebutape originate from CuDerm Corporation (Dallas, TX, USA).

### Group study

A total of 101 subjects from Beijing Obstetrics and Gynecology Hospital, Capital Medical University (Beijing, China) were selected for this study (53 second-trimester women, age: 30.58 ± 4.10 years, gestational weeks: 20.08 ± 4.74 weeks, 38 first-time pregnancies; and 48 controls, age: 30.28 ± 5.54 years). All volunteers were healthy and had no other systemic or skin problems. The purpose of this experiment was disclosed to all volunteers, who subsequently signed informed consent forms. This is a non-invasive study and followed all principles of the Declaration of Helsinki. This study was reviewed by the Medical Ethics Committee of Beijing Obstetrics and Gynecology Hospital, Capital Medical University.

### Sample collection and preparation

Lipid samples were collected from the cheeks (the most prominent area of the cheekbones on either side) of volunteers using Sebutape according to our previous study^18^. Sample pre-processing was performed using the Bligh and Dyer method^18^.

### UPLC-QTOF-MS analysis and data extraction

UPLC-QTOF-MS equipment and experimental conditions were consistent with our previous work. The data collection and analysis methods also referred to previous studies on acne^18,19^. The above experimental instruments and data analysis software were obtained from Waters Corporation (Milford, MS, USA). Comparisons were made using the LIPID MAPS Structure Database (LMSD) (http://www.lipidmaps.org/) to determine the compound ID of the lipid composition of each sample.

### Data analysis

The raw data were processed using Progenesis QI 2.0, and then transfered to Ezinfo 3.0 for principal component analysis (PCA) and orthogonal projections to latent structures discriminant analysis (OPLS-DA). Pareto was used for data scaling and centring. The selection criteria were variable influence on projection (VIP) > 3, *p *value < 0.05, and fold change > 2 for selecting the most important entities. The significance of biological parameters differences between group was determined using IBM SPSS Statistics 19.0 software (IBM SPSS, Armonk, NY, USA) for the Student's *t*-test by Excel 2016 (Microsoft, Redmond, WA, USA). *p* < 0.05 was considered significant, and *p* < 0.001 was considered highly significant. For all analyses, *** *p* < 0.001; ** *p* < 0.01; * *p* < 0.05.

## Results

### PCA and OPLS‐DA of facial lipids

**Figure 1:**
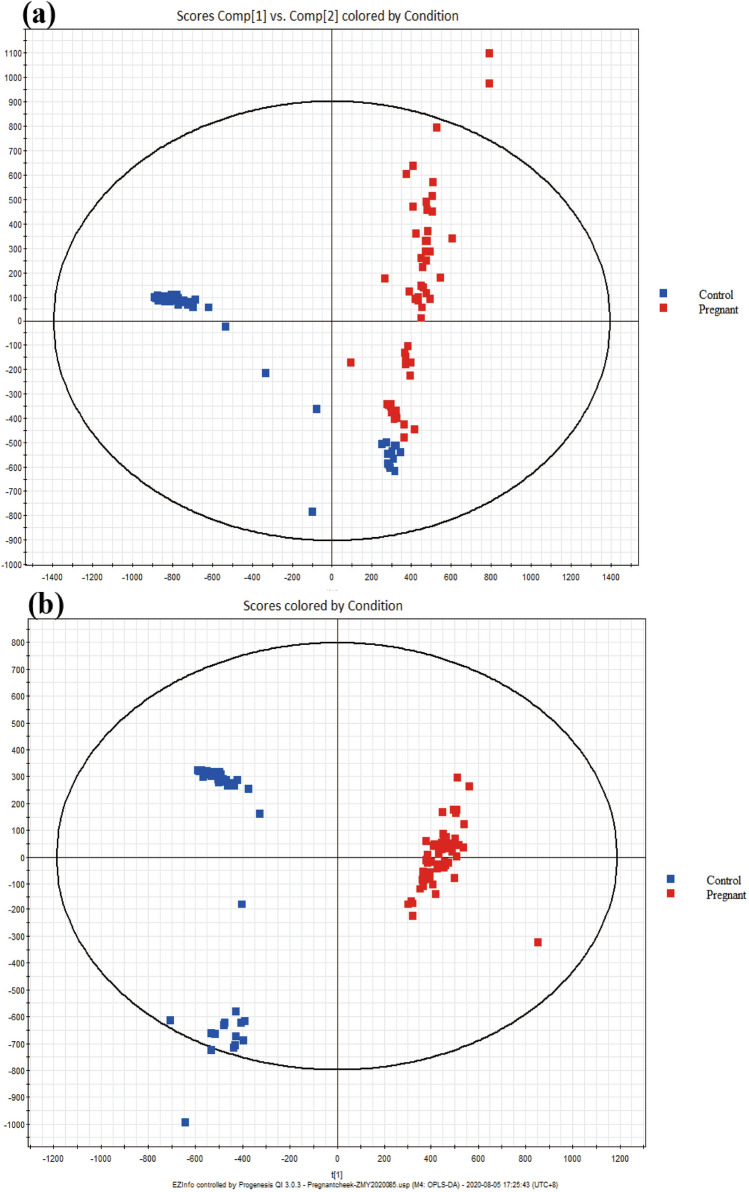
(**a**) Principal component analysis (PCA) score plot of skin surface lipids (SSL) composition between pregnant women and non-pregnant women. (**b**) Orthogonal projections to latent structures discriminant analysis (OPLS-DA) score plot of SSL composition between pregnant women and non-pregnant women.

SSLs from the pregnant and control groups were analysed using untargeted lipidomics based on the fine stability of UPLC-QTOF-MS. PCA and OPLS-DA models were used for multivariate data analysis. The resulting model score plot is shown in Fig. 1. These results demonstrated good separation and significant differences between the pregnant and control SSL samples.

### Variations in the eight main classes of lipids

Lipid entities were analysed based on differences between the two groups. The results identified 2270 lipids and separated them into eight major classes.

**Figure 2:**
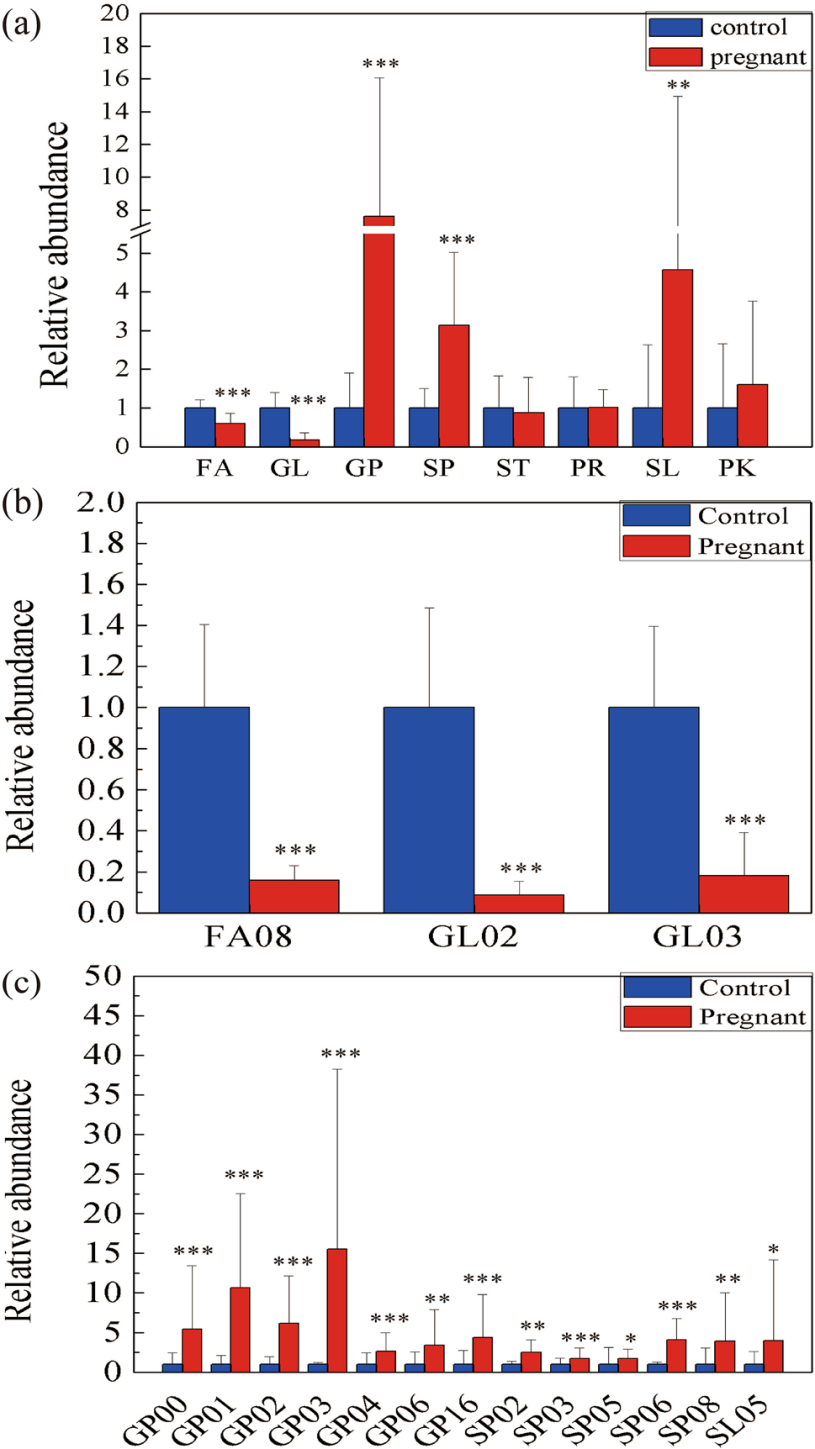
(**a**) The relative amounts of the eight major lipids in the pregnant and control groups. (**b**) 3 subclasses in fatty acyls (FA) and glycerolipids (GL) of the relative amounts significantly decreased. (**c**) 13 subclasses in 3 main classes (glycerophospholipids (GP), sphingolipids (SP), and saccharolipids (SL)) of the relative amounts significantly increased. ***p < 0.001, **p < 0.01, *p < 0.05.

We calculated the relative content of each major lipid class. Figure 2a illustrates the differences between the eight classes by showing the relative content of each major lipid class in the two groups. In the pregnancy group, two main classes significantly (*p* < 0.05) decreased (fatty acyls [FAs] and glycerolipids [GLs]), while three other classes (glycerophospholipids [GPs], sphingolipids [SPs], and saccharolipids [SLs]) significantly increased (*p* < 0.05). The remaining three classes (sterol Lipids [STs], prenol lipids [PRs], and polyketides [PKs]) were not significantly different between groups. The corresponding relative abundance values and lipid species information are shown in Table S1.

### Relative content alterations in lipid subclasses

Analyses were then performed to identify the subclasses responsible for the significant changes in the five main classes mentioned above. The relative changes in subclasses from the five major classes were analysed. In the SSL profile of pregnant women, 16 subclasses differed significantly (*p* < 0.05), displaying the same change trend as the major class to which they belonged. As shown in Fig. 2b,c, the relative amounts of three subclasses (fatty amides [FA08], diradylglycerols [GL02], triradylglycerols [GL03]) from 2 main classes (FAs and GLs) significantly decreased (*p* < 0.05), and the relative amounts of 13 subclasses (other glycerophospholipids [GP00], glycerophosphoethanolamines [GP02], glycerophosphoserines [GP03], glycerophosphoglycerols [GP04], glycerophosphoinositols [GP06], glycerophosphonocholines [GP16], ceramides [SP02; Cers], phosphosphingolipids [SP03], neutral glycosphingolipids [SP05], acidic glycosphingolipids [SP06], amphoteric glycosphingolipids [SP08], and other acyl sugars [SL05]) from 3 main classes (GPs, SPs, and SLs) significant increased (*p* < 0.05).

### Identification of important individual lipids in the pregnant and control groups

**Figure 3:**
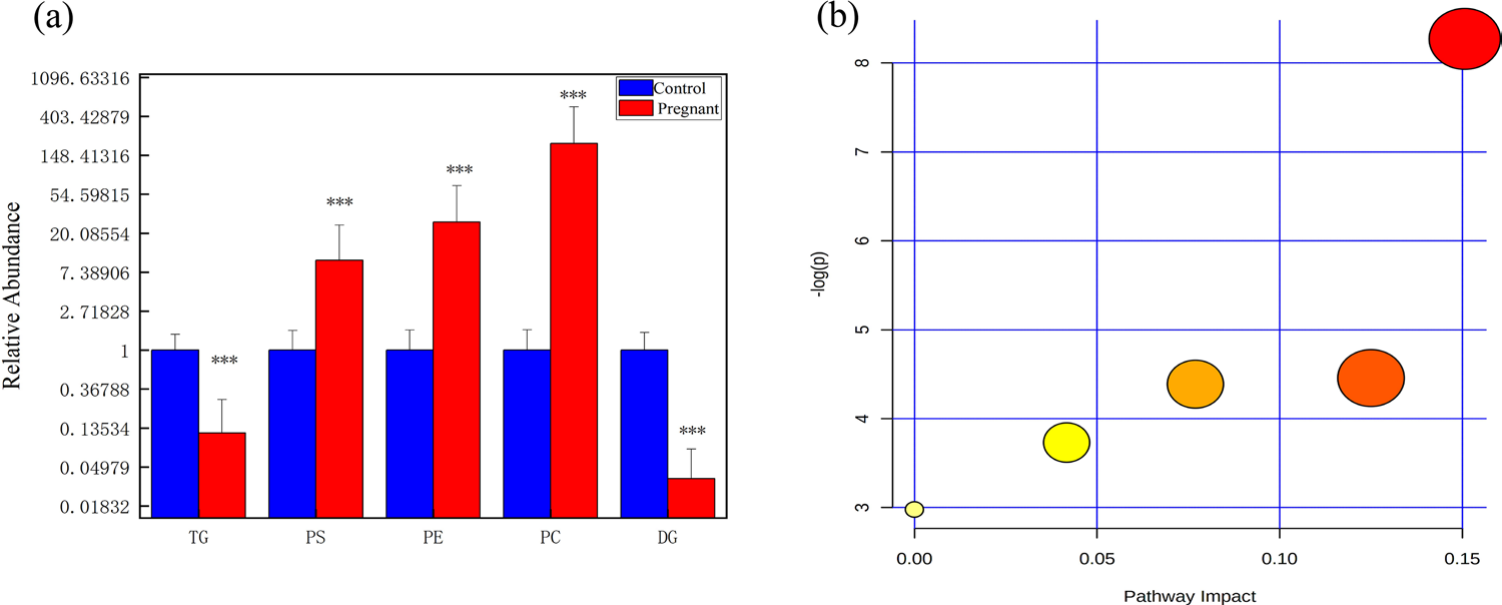
(**a**) Relative levels of important individual lipids in responsible for differentiating between the pregnant and control groups. TG, triglyceride. PS, phosphatidylserine. PE, phosphatidylethanolamines. PC, phosphatidylcholine. DG, diacylglycerol. (**b**) Metabolic pathway maps of important individual lipids responsible for differentiating between the pregnant and control groups. From left to right, the metabolic pathways in order are glycosylphosphatidylinositol (GPI)-anchor biosynthesis, linoleic acid metabolism, alpha-Linolenic acid metabolism, arachidonic acid metabolism, and glycerophospholipid metabolism.

OPLS-DA was applied to identify the most important lipid individuals within the aforementioned subclasses, which were responsible for distinguishing pregnant samples from controls. The selection criteria were as follows: VIP > 3, *p* < 0.05, and fold change > 2. As shown in Table 1, 23 lipids were identified (for more information on the MS/MS spectra and ion fragments of characteristic lipids, see Fig. S1). Among them, the phosphatidylserine (PS), phosphatidylethanolamine (PE), and phosphatidylcholine (PC) contents were increased in the pregnancy group, and the triglyceride (TG) and diacylglycerol (DG) contents were decreased in the pregnancy group (Fig. 3a).

**Table 1:** Screened individual skin surface lipids (SSL) lipids most important for distinguishing between the pregnant and control groups.

m/z	Formula	Description	Anova (*p*)	Highest mean	Adducts
559.517	C18H33NO	Linoleamide	0.000	Control	M + Na
603.534	C39H70O4	1-(14-methyl-pentadecanoyl)-2-(8-[3]-ladderane-octanyl)-sn-glycerol	0.000	Control	M + H
647.558	C41H74O5	DG(18:3(9Z,12Z,15Z)/20:0/0:0)[iso2]	0.000	Control	M + H
684.193	C30H35O18+	Petunidin 3-glucoside-5-(6″-acetylglucoside)	0.004	Pregnant	M + H
794.497	C43H72NO10P	PS(20:5(5Z,8Z,11Z,14Z,17Z)/17:1(9Z))	0.000	Pregnant	M + H
808.506	C42H76NO10P	PS(18:3(9Z,12Z,15Z)/18:0)	0.001	Pregnant	2 M + H
852.556	C50H78NO8P	PC(20:5(5Z,8Z,11Z,14Z,17Z)/22:6(4Z,7Z,10Z,13Z,16Z,19Z))	0.000	Pregnant	M + Na
855.741	C55H98O6	TG(16:1(9Z)/18:0/18:3(9Z,12Z,15Z))[iso6]	0.000	Control	M + Na
862.539	C49H78NO8P	PE(22:6(4Z,7Z,10Z,13Z,16Z,19Z)/22:4(7Z,10Z,13Z,16Z))	0.000	Pregnant	M + Na
866.567	C49H82NO8P	PE(22:6(4Z,7Z,10Z,13Z,16Z,19Z)/22:2(13Z,16Z))	0.002	Pregnant	M + H
874.785	C54H97D5O6	TG(17:0/17:1(10Z)/17:0) (d5)	0.000	Control	M + H
877.726	C57H96O6	TG(14:1(9Z)/18:0/22:6(4Z,7Z,10Z,13Z,16Z,19Z))[iso6]	0.000	Control	M + Na
878.565	C52H80NO8P	PC(22:6(4Z,7Z,10Z,13Z,16Z,19Z)/22:6(4Z,7Z,10Z,13Z,16Z,19Z))	0.000	Pregnant	M + H
878.565	C50H82NO8P	PC(20:5(5Z,8Z,11Z,14Z,17Z)/22:4(7Z,10Z,13Z,16Z))	0.000	Pregnant	M + H
879.741	C57H98O6	TG(16:1(9Z)/18:0/20:5(5Z,8Z,11Z,14Z,17Z))[iso6]	0.000	Control	M + H
880.579	C50H84NO8P	PC(22:6(4Z,7Z,10Z,13Z,16Z,19Z)/20:2(11Z,14Z))	0.000	Pregnant	M + H
881.760	C57H100O6	TG(16:1(9Z)/18:0/20:4(5Z,8Z,11Z,14Z))[iso6]	0.000	Control	M + Na
883.770	C55H104O6	TG(15:0/18:0/19:1(9Z))[iso6]	0.000	Control	M + Na
899.717	C57H96O6	TG(14:1(9Z)/18:0/22:6(4Z,7Z,10Z,13Z,16Z,19Z))[iso6]	0.000	Control	M + Na
903.742	C59H98O6	TG(14:1(9Z)/20:1(11Z)/22:6(4Z,7Z,10Z,13Z,16Z,19Z))[iso6]	0.000	Control	M + Na
905.757	C59H100O6	TG(14:1(9Z)/20:0/22:6(4Z,7Z,10Z,13Z,16Z,19Z))[iso6]	0.000	Control	M + Na
909.785	C57H106O6	TG(17:1(9Z)/18:0/19:1(9Z))[iso6]	0.000	Control	M + H, M + Na
909.787	C59H104O6	TG(14:1(9Z)/20:0/22:4(7Z,10Z,13Z,16Z))[iso6]	0.000	Control	M + Na

*TG* triacylglyceride, *PC* phosphatidylcholine, *PS* phosphatidylserine, *PE* phosphatidylethanolamines, *DG* diacylglycerol.

### Metabolic pathway analysis

The lipids from Table 1 were imported into MetaboAnalyst 4.0 (http://www.metaboanalyst.ca/) to explore the metabolic pathways for SSL changes during pregnancy. The metabolic pathway analysis module identified five metabolic pathways associated with skin conditions during pregnancy (Fig. 3b). These metabolic pathways included GP metabolism, glycosylphosphatidylinositol (GPI)-anchor biosynthesis, arachidonic acid metabolism, linoleic acid metabolism, and alpha-linolenic acid metabolism. GP metabolism had the largest impact factor and largest circle in the upper right corner, so it was considered the most important metabolic pathway. This pathway included the metabolic pathways involved in PC and PE.

### Average ceramides (Cers) and fatty acids (FAs) chain length

**Figure 4:**
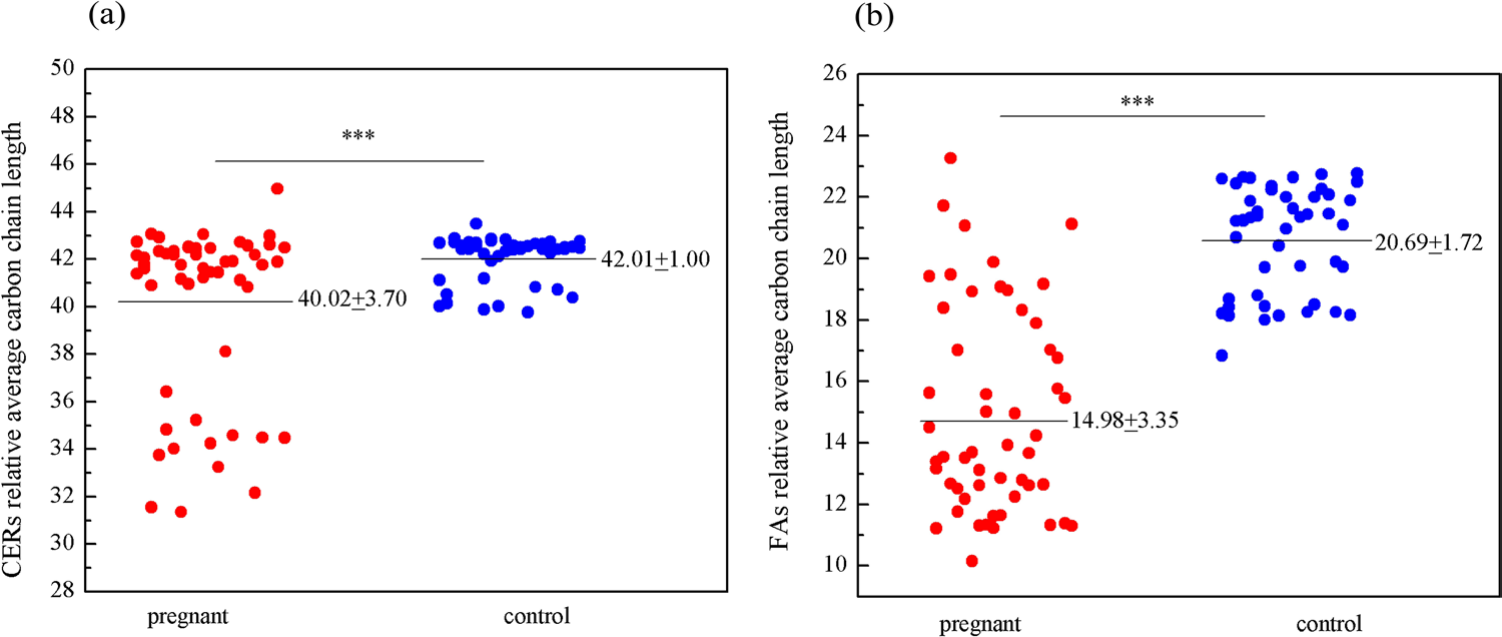
(**a**) Ceramide (Cers) relative average carbon chain length in patients with pregnant compared with control. (**b**) Fatty acyls (FAs) relative average carbon chain length in patients with pregnant compared with control.

It is well known that the long acyl chain in Cers is necessary to form tightly wrapped impermeable lipid lamellae. The skin barrier has a high affinity for the chain lengths of Cer and FAs^20–22^. We observed a significant reduction in mean Cer and FA chain length in the pregnancy group compared to the control group (Fig. 4). The relevant Cer subclasses sre shown in Table S2. In the pregnancy group, significantly decreased levels of unsaturated free fatty acids (FFAs) and saturated FFAs increased, but no significant changes were observed. In conclusion, the observed changes in lipid composition as well as reductions in Cer, and FFA chain lengths in the pregnancy group contributed to the altered lipid organization and reduced skin barrier function.

## Discussion

This study was conducted to confirm whether changes in the skin barrier during pregnancy is associated with SSL changes. Research has shown that approximately 60–80% of women first develop eczema during pregnancy, and over 50% experience skin deterioration^23^. An impaired skin barrier, which increases skin susceptibility and, thereby results in immune activation, has been documented in atopic eczema^24,25^. Trans Epidermal Water Loss (TEWL) refers to the total amount of water loss through the skin, which can be used as an index of skin barrier function for non-invasive measurement. A previous study found that TEWL increases in pregnancy and the postnatal period^6^. Lipids significantly affect the skin state through different mechanisms, such as maintaining the barrier properties of the skin and acting as signaling molecules to transmit intercellular signals^7,26^. Lipidomics employ mass spectrometry to analyse and characterise lipid species in biological systems on a large scale^18,19,27^. In this study, we used lipidomics method to study the changes of lipids on the facial skin surface of women in the second trimester. The study found significant lipid changes on the skin surface of the pregnancy group (Fig. 2), which may be related to the fragile skin barrier of pregnant women.

Overall, the total SSL content in the pregnancy group was lower and significantly different than that in the control group (*p* < 0.01), presumably due to the weakened skin barrier function during pregnancy (see Figure S2). We identified a total of 2270 lipids and found that among the eight main classes of lipids, the relative abundances of GPs, SPs, and SLs increased significantly in the pregnancy group while those of FAs and GLs decreased significantly. Based on the significant trends in these five main classes of lipids, we screened 16 subclasses of lipids and analysed them under the screening criteria of VIP > 3, *p* < 0.05, and fold change > 2. Twenty-three individual lipid species that contributed significantly to the group differences were selected, and PC was the most abundant lipid in the pregnancy group. The obtained GP metabolic pathway included two lipids, PC and PE, both of which made important contributions to the group differences. Furthermore, we observed a decrease in Cers average chain length and an increase in FFA in the SSL profile of the pregnancy group compared to that of the control group.

Women have the highest levels of serum ostrogen during pregnancy. In normal pregnancy, the female body produces large amounts of ostrogen and progesterone. The concentrations of serum estrone and oestradiol increases by approximately 50-fold compared to their maximum pre-pregnancy values, and estriol increases by approximately 1000-fold^28^. Because of these changes during pregnancy, the skin becomes more sensitive and fragile due to increased sensitivity to external stimuli^29^. Topical ostrogen is known to inhibit sebum production, and ostrogen-containing contraceptives are used in the treatment of female acne^30^. Loss of ostrogen after menopause has been shown to lead to reduced sebum production^31^. There are several hypotheses that elucidate the mechanism by which ostrogen inhibits sebum production. These include the idea that ostrogen directly antagonizes androgenic activity, regulates genes associated with lipogenesis, and inhibits androgen production by gonadal tissue via a negative feedback loop^32^. Both receptors for ostrogen, ERα and Erβ, are expressed in basal and differentiated sebaceous cells, suggesting that the site of ostrogen action in the sebaceous gland is quite important^33^. In a study monitoring the skin condition at all stages of the menstrual cycle, it was found that blood progesterone levels peaked between days 15 and 28 while the skin barrier was weakest between days 22 and 26^34^. An early study found a significant decrease in sebum secretion in the middle of gestation (28–32 weeks) with slight fluctuations throughout the pregnancy, which is consistent with our results^35^. Therefore, we speculate that due to changes in ostrogen during pregnancy, there is a decrease in SSL secretion and weakening of the skin barrier. However, some epidemiological investigation^4,36–38^ have found that acne is also a common condition during pregnancy. It is worth noting that acne occurs most often in the third trimester of pregnancy due to increased sebum secretion from maternal androgens, which does not contradict our results (the sampling population was women in the second trimester of pregnancy).

GP metabolism is significantly affected in a variety of diseases associated with pregnancy, such as preeclampsia^39^, recurrent miscarriage^40^, placental syncytiotrophoblast microvesicles in adverse pregnancy^41^, and early in utero growth restriction of breast milk and macrosomic babies^42^, which indicates that the process of pregnancy may cause a disturbance in the metabolism of GPs. Some GPs are involved in signaling pathways for cell survival and inflammation and affect membrane-associated proteolysis^43^. Alterations in GP metabolism also occur in psoriasis and lead to defects in skin fibroblasts and breakdown of the skin barrier in patients with neutral lipid storage disease with ichthyosis^44,45^.

Notably, the screened differential lipids PC, PE are also involved in the metabolism of GPs, and the PC content was the highest among the 23 differential lipids. PC and PE are the main phospholipid components of the cell membrane, with PC accounting for 50% of the total phospholipids and PE content comprising approximately 20–30%^46^. PC is the main phospholipid in mammalian cell membranes and plays important roles in necrosis, signal transduction, protein sorting and apoptosis^47^. PC is the primary source of many second messengers (such as arachidonic acid and DAG) and is often further metabolized into other signaling elements^48^. PC also serve as a mitogen needed for growth factors-induced DNA synthesis^48^. PE is often involved in oxidative phosphorylation, membrane fusion, and mitochondrial biogenesis and is sensitive to inflammatory stimuli^49,50^. PE also plays a pivotal role in cell division to ensure proper cytoplasmic division. PE-rich domains are exposed at the cleavage groove on the cell surface to regulate interactions between the contractile ring and plasma membrane^51^. In the final stages of cytoplasmic division in some cells, PE appears to be required for the breakdown of actin filaments; in other words, cells that lack PE cannot complete division and separation^52^. It has been found that PC and PE contents increase under particulate matter 2.5 exposure, resulting in metabolic disorders of related lipids and thereby inducing inflammatory reactions and atherosclerosis acceleration^53^. In addition, we found that the ratio of PC/PE was significantly higher in the pregnancy group than in the control group. Studies have found that malignant tumor cells with metastasis have a higher PC/PE ratio than those without metastasis^54^. This is associated with increased synthesis of cell membranes and accelerated replication of tumor cells. It has also been shown that PC, PE, and PC/PE ratios are significantly higher in prostate cancer tissue than in benign prostate tissue^55^. Based on the above discussion, we speculate that an increase in PC and PE contributes to the growth and division of skin cells during pregnancy while also being more sensitive to inflammation. Thus, the PC/PE ratio increase during pregnancy may be associated with accelerated membrane integrity as well as increased energy generation.

Interestingly, compared to our previous experiments, in the study of SSL differences between men and women, women had higher levels of GPs, such as PG, PC, PE, PS, PA, and PI^17^. The levels of PC, PE, and PS in this study were higher in pregnant women than in non-pregnant women. This finding appears to indicate that the levels of certain GPs are correlated with female status, which may be related to ostrogen levels in women; however, further experiments are needed to verify the hypothesis.

During pregnancy, the mother’s immune response is extensively altered to allow the foetus to better attach to the mother^56^. Hormonal changes during pregnancy also alter the cytokine profile, resulting in a preference for Th2-type cytokines (interleukin [IL]-4, IL-5, IL-10, and IL-13) that contribute to the maintenance of fetal survival^3^. This local immune change appears to translate into peripheral effects. Studies have shown that the levels of both regulatory T cell cytokines (IL-10 and sTNFRII) and Th1-type cytokines (IL-2, IL-12, and IL-27) are elevated in mid-pregnancy^57^. Alterations in immunity will also affect the synthesis and proportion of lipids. IL-13 inhibits FFA elongases (Elongation of very long chain fatty acids [ELOVL] 3 and 6) in a STAT6-dependent manner and downregulates expression in mouse skin lesions^58^. IL-4 downregulates mRNAs encoding sphingomyelinase (SMase) and glucocerebrosidase (GBA) and upregulates mRNA encoding acid ceramidases, resulting in lower Cer levels^59^. Th2 cytokines significantly reduce the expression levels of the epidermal lipid metabolism enzyme-encoding genes *SMase*, *GBA* and *Elovl1*, resulting in significant downregulation of specific Cer levels^60^. These immune factor-induced changes in lipids are closely associated with increased TEWL and AD phenotypes^61,62^. Therefore, we inferred that lipid disturbance along with alterations in the immune system work together to weaken the immune and physical barriers of the skin during pregnancy.

We also observed a decreased Cer chain length in the pregnancy group, which was consistent with our previous research on acne^18,19^, as well as a decrease in FA chain length, which is also reflected in adolescent acne patients^63^, a slight decrease in unsaturated FFAs content, and a slight increase in saturated FFAs content. When long-chain Cers decrease and short-chain Cers increase, the skin barrier is known to become defective^58^. Cers are the main lipids in the stratum corneum, accounting for approximately one-third of lipid molecules or 50% of their weight. Cers contain a large number of hydrophilic groups, which have a good affinity for water. Cer promote epidermal hydration and enhance epidermal cell cohesion. They also form a bedplate bilayer structure in the keratinocytes, which effectively prevents epidermal water from escaping. Cer can penetrate deep into the epidermis, prompting the skin to regain the ability to retain water, so they have an outstanding moisturizing effect. In addition, Cers can initiate aging cells, promote epidermal cell division and basal layer cell regeneration, enhance the role of the skin barrier (or isolation protection), and prevent the invasion of external irritants. Although FA carbon chains were reduced in the pregnancy group, the FA content did not increase (Figure S2), suggesting that the reduction in carbon chains was not used for oxidation reactions and that long-chain FA were the majority of the contents in both groups. Meanwhile, the long-chain FA content in the pregnancy group was significantly lower than that in the control group (*p* < 0.5). Long-chain FAs protect the body as a component of the skin barrier. FAs are extended by the addition of two carbons through a four-step process (condensation, reduction, dehydration, and reduction), and the rate-limiting step of the FA elongation cycle is a condensation step catalyzed by seven elongases (Elovl1to-7). Li et al.^64^ found that Elovl4-deficient mice were deficient in long-chain FA-derived Cers, resulting in defective skin permeability barrier function and neonatal mortality. Mice with Elovl1 gene deletion exhibited severe epidermal barrier defects and died shortly after birth. In these mice, lipid lamellar formation was largely impaired^65^. In addition, Cer in the epidermis contains extremely long FAs. Reduced levels of epidermal Cer and changes in FA chain length can lead to several skin diseases. Elovl1 is a key determinant of epidermal Cer chain length and is differentially regulated in a tissue-specific manner^65^. This explains the commonality between the changes in FA chain length and Cer chain length, which collectively weaken the skin barrier. An early study showed that FA chain length affects the phase behavior of cholesterol/Cer/FA mixtures, and the lipid phase behavior of the three lipid mixtures is very similar to that of the intact cuticle with only long-chain FFAs^66^. The decrease in the average chain length of Cer and the decrease in the expression of FA synthetase (e.g. elongase) leads to a significant shortening of the FFA chain length, which is also reflected in AD^67^. When long-chain FAs are replaced by short-chain FAs in the intercellular space of the healthy human stratum corneum, the laminar structure of the skin tissue is affected, resulting in skin barrier dysfunction. When the FFAs in lipid mixtures with simulated sebum fractions are changed from short-chains to long-chains, the mixtures become more structurally robust, and their stability is enhanced. In addition, studies have shown that FFAs can bind to newly synthesized PC and PE and FFA are important drivers of PC synthesis in human skin fibroblasts^68^. These results further corroborate that lipid disorders weaken the skin barrier in women during pregnancy.

Current research on pregnant women has focused on pigmentation, pruritus and changes in plasma lipids. For the first time, we investigated lipid changes in the facial skin of pregnant women and found significant differences in lipid composition compared to that of the non-pregnant control group, presumably related to GP metabolism. Although most skin changes subside after pregnancy, the weakening skin barrier during pregnancy calls for greater attention to skin care and careful cosmetic product selection. Our study provides insights for better skin management during pregnancy.

## Supplementary Information

Supplementary Information.

Supplementary Table.
