# Supplementary material for: Lipidomic analysis of facial skin surface lipid reveals the causes of pregnancy-related skin barrier weakness
Source: Sci Rep. 2021 Feb 5;11:3229. doi: 10.1038/s41598-021-82624-3 (PMC7864992; doi:10.1038/s41598-021-82624-3)
Supplement: Supplementary file 1 — Supplementary Information. [file 41598_2021_82624_MOESM1_ESM.docx]

**Lipidomic analysis of facial skin surface lipid reveals the causes of** **pregnancy-related skin barrier weakness**

Manli Yang,^1,2^ Mingyue Zhou,^3^ Yuan Li, ^4^ Hong Huang, ^4^ Yan Jia,^1,2#^

**Current affiliations and addresses:**

^1^Beijing Key Laboratory of Plant Resources Research and Development, College of chemistry and materials engineering, Beijing Technology and Business University, Beijing, China

^2^Key Laboratory of Cosmetic of China National Light Industry, College of chemistry and materials engineering, Beijing Technology and Business University, Beijing, China

^3^Chongqing Key Laboratory of Translational Research for Cancer Metastasis and Individualized Treatment, Chongqing University Cancer Hospital, Chongqing, China

^4^Department of Gynecology, Beijing Obstetrics and Gynecology Hospital, Capital Medical University, Beijing, China

***Correspondence:** Yan Jia, Beijing Key Laboratory of Plant Resources Research and Development, Key Laboratory of Cosmetic of China National Light Industry, College of chemistry and materials engineering, Beijing Technology and Business University, Beijing 100048, China. Tel.: +86 10 68984937, E-mail: jiayan@btbu.edu.cn.

Contents:

Fig. S1 MS, MS/MS spectra, and ion fragments of characteristic lipids

Fig. S2 Total SSL content in the pregnant group and the healthy group.

Table S1 Relative abundance values and lipid species for identified 2270 lipids.

Table S2 The relevant CER subclasses.


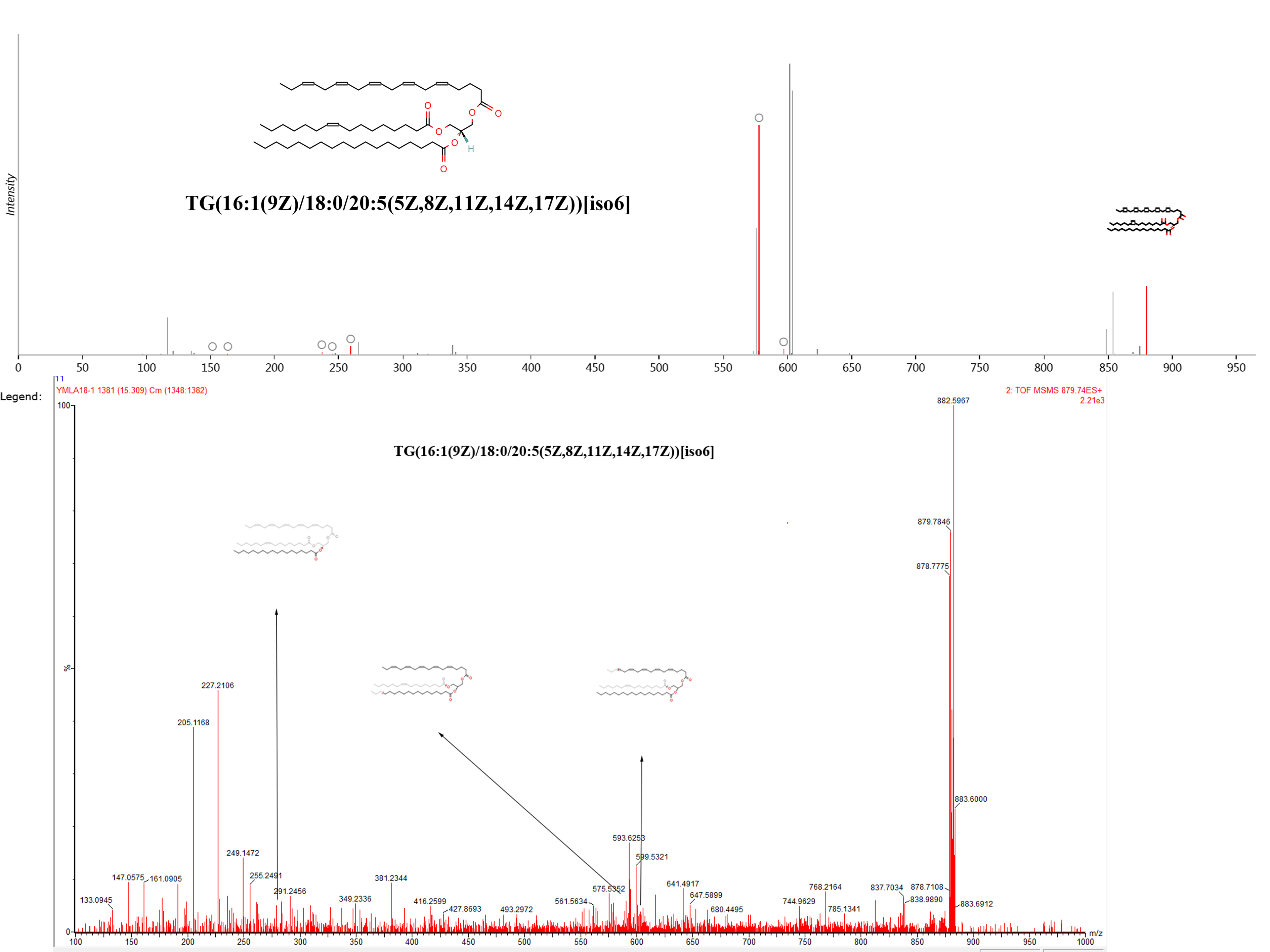


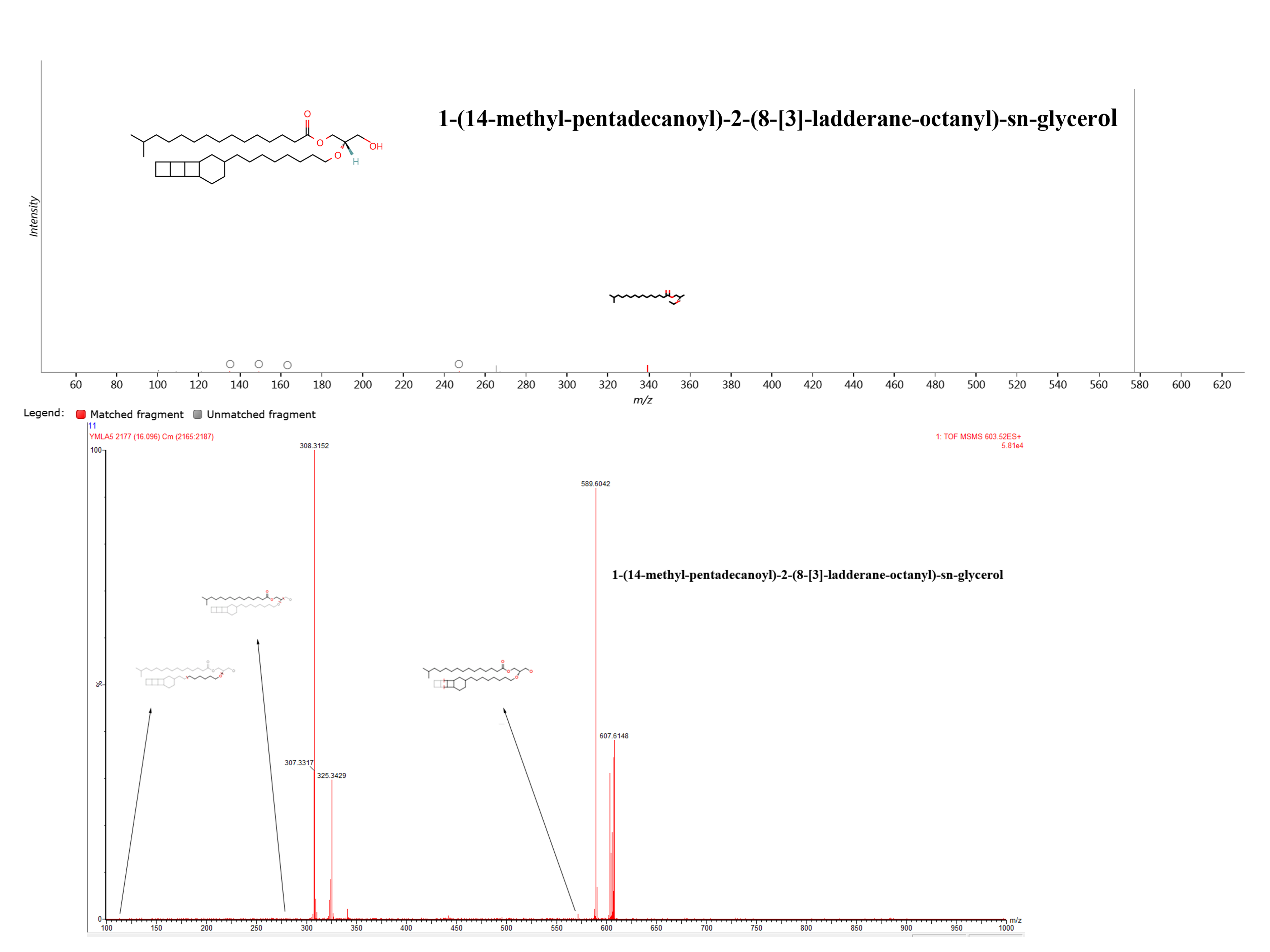


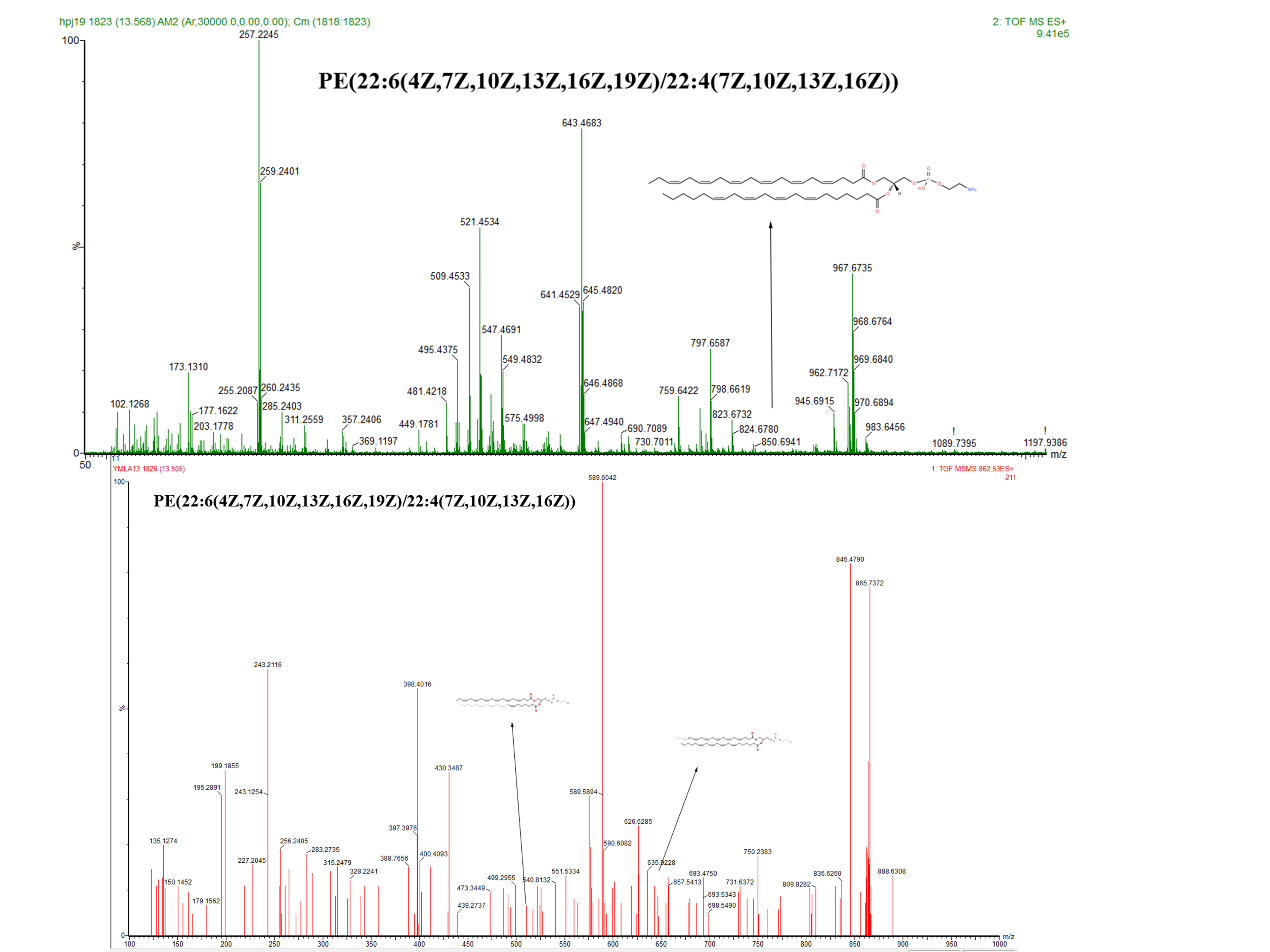


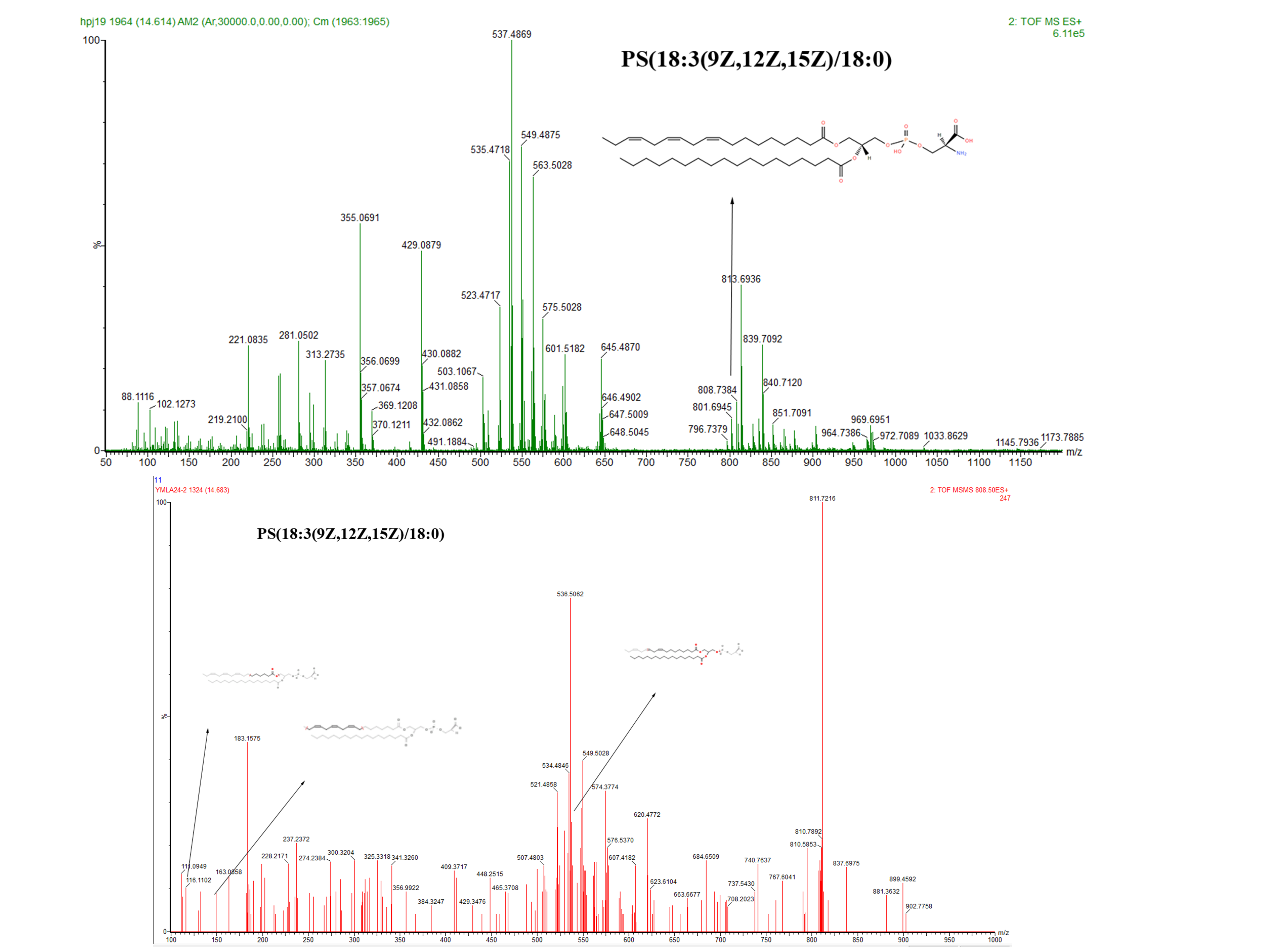


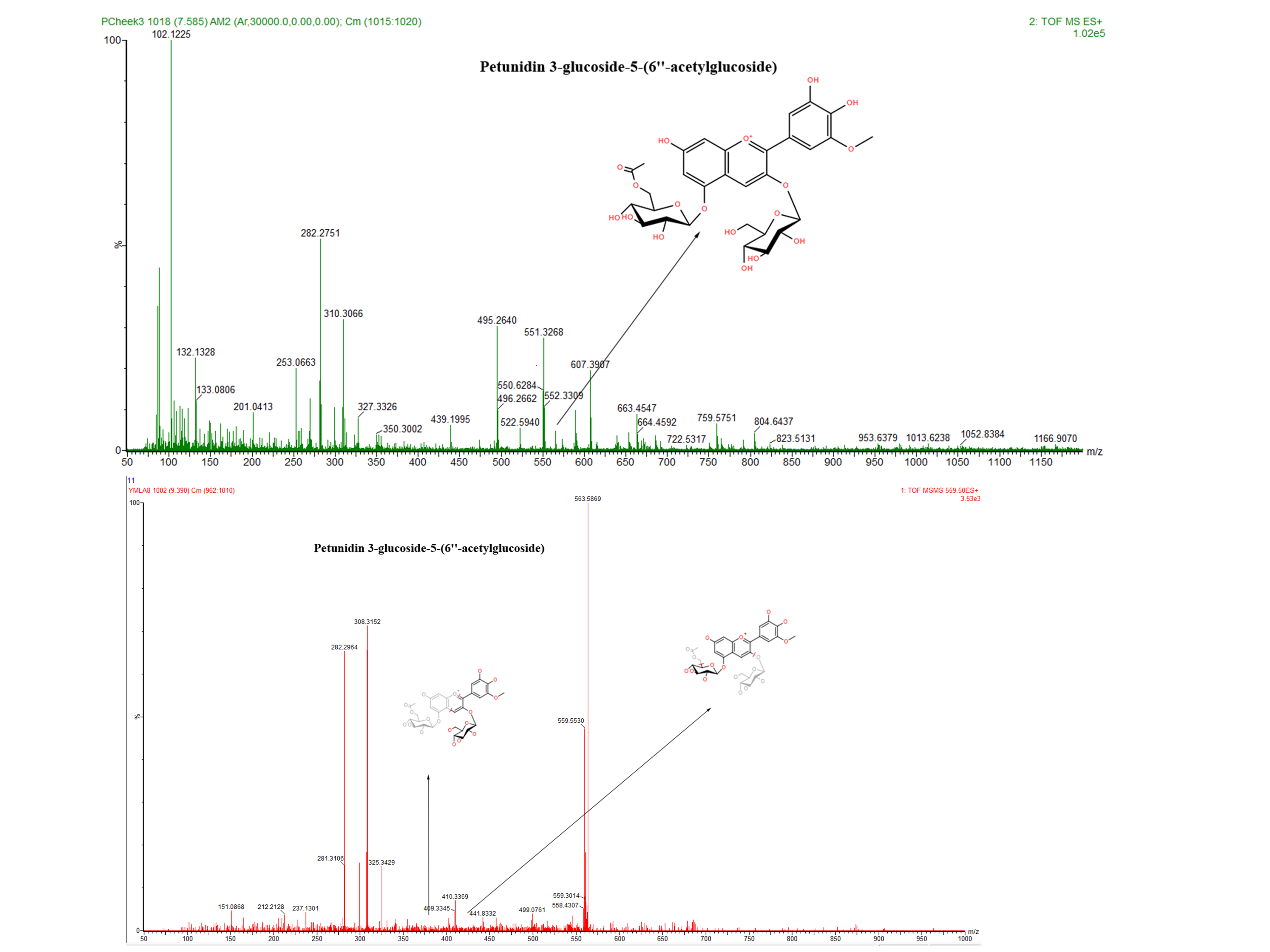


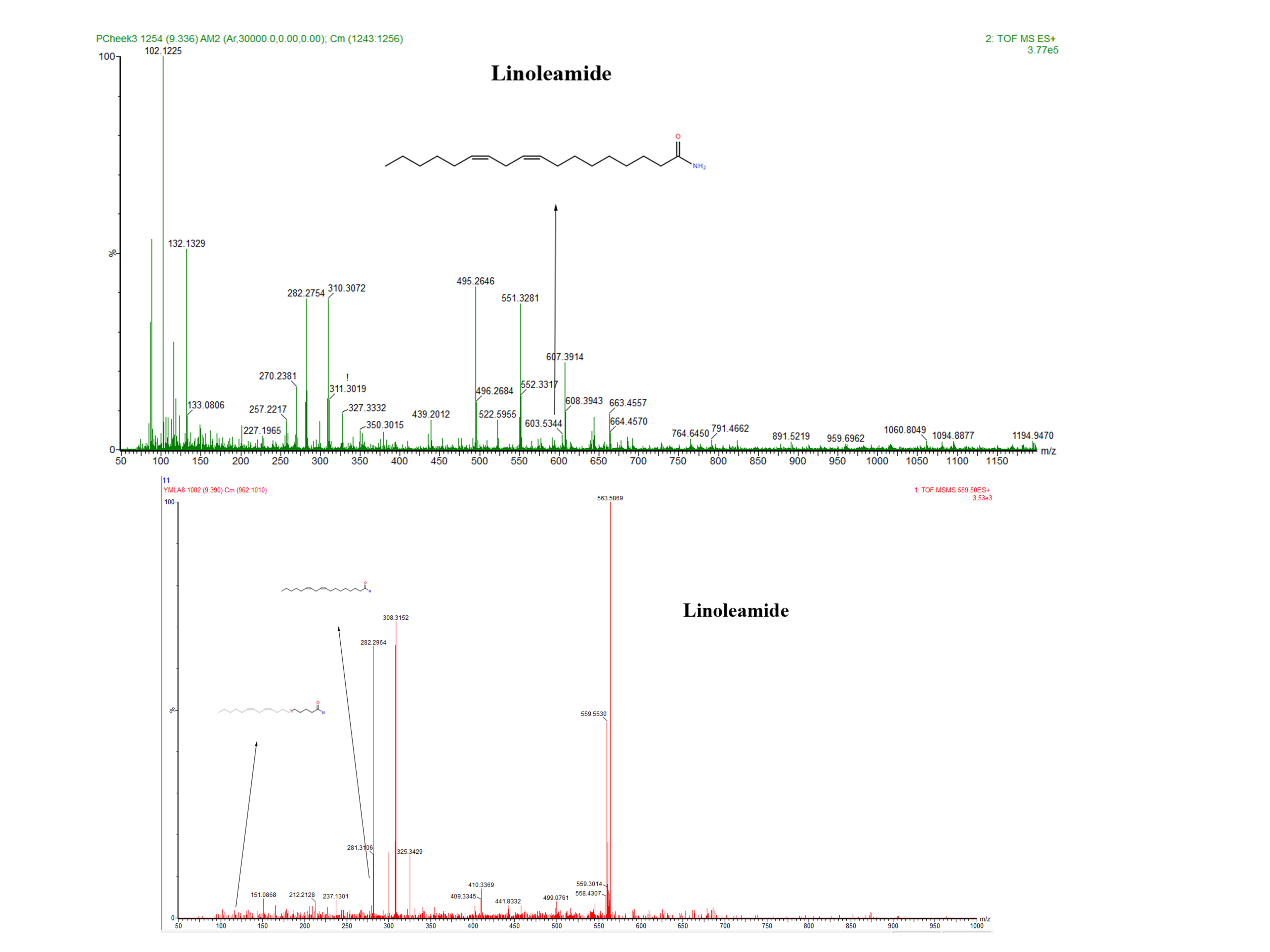


Fig. S1 MS, MS/MS spectra, and ion fragments of characteristic lipids


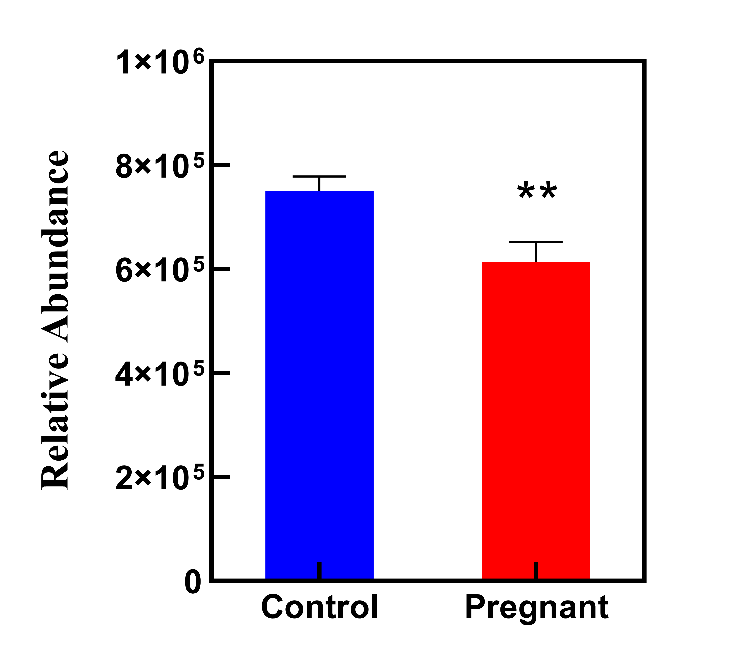


Fig. S2 Total SSL content in the pregnant group and the healthy group.

Table S1 Relative abundance values and lipid species for identified 2270 lipids.

For the table is too large so it is put in a separate excel.

Table S2 The relevant CER subclasses.

| **Compound ID** | **Formula** | **Description** | **m/z** | **Retention time (min)** |
| --- | --- | --- | --- | --- |
| **LMSP02010002** | **C30H59NO3** | **Cer(d18:1/12:0)** | **504.440** | **6.045** |
| **LMSP02050001** | **C30H60NO6P** | **CerP(d18:1/12:0)** | **584.407** | **1.029** |
| **LMSP02010001** | **C32H63NO3** | **Cer(d18:1/14:0)** | **510.487** | **6.720** |
| **LMSP02010023** | **C33H63NO3** | **Cer(d18:2/15:0)** | **1043.971** | **10.946** |
| **LMSP02010019** | **C33H67NO3** | **Cer(d18:0/15:0)** | **548.503** | **9.573** |
| **LMSP02010024** | **C34H65NO3** | **Cer(d18:2/16:0)** | **1072.003** | **7.723** |
| **LMSP02010004** | **C34H67NO3** | **Cer(d18:1/16:0)** | **538.517** | **9.317** |
| **LMSP02050002** | **C34H68NO6P** | **CerP(d18:1/16:0)** | **640.467** | **1.556** |
| **LMSP02030001** | **C34H69NO4** | **Cer(t18:0/16:0)** | **556.530** | **10.214** |
| **LMSP02010020** | **C35H69NO3** | **Cer(d18:1/17:0)** | **574.519** | **6.585** |
| **LMSP02010031** | **C35H71NO3** | **Cer(d18:0/17:0)** | **576.532** | **8.962** |
| **LMSP02020019** | **C35H71NO4** | **Cer(d18:0/h17:0)** | **570.545** | **10.91** |
| **LMSP02010006** | **C36H71NO3** | **Cer(d18:1/18:0)** | **566.551** | **16.914** |
| **LMSP02050004** | **C36H72NO6P** | **CerP(d18:1/18:0)** | **668.498** | **2.168** |
| **LMSP02020008** | **C36H73NO3** | **Cer(d18:0/18:0)** | **568.563** | **6.799** |
| **LMSP02010032** | **C37H73NO3** | **Cer(d18:1/19:0)** | **580.565** | **3.285** |
| **LMSP02010016** | **C38H75NO3** | **Cer(d16:1/22:0)** | **594.579** | **10.811** |
| **LMSP02050005** | **C38H76NO6P** | **CerP(d18:1/20:0)** | **696.527** | **2.303** |
| **LMSP02020009** | **C38H77NO3** | **Cer(d18:0/20:0)** | **596.595** | **8.798** |
| **LMSP02010017** | **C39H77NO3** | **Cer(d16:1/23:0)** | **608.595** | **10.840** |
| **LMSP02010029** | **C40H77NO3** | **Cer(d18:2/22:0)** | **642.577** | **15.286** |
| **LMSP02010008** | **C40H79NO3** | **Cer(d18:1/22:0)** | **622.611** | **10.491** |
| **LMSP02020010** | **C40H81NO3** | **Cer(d18:0/22:0)** | **624.631** | **6.550** |
| **LMSP02010030** | **C41H79NO3** | **Cer(d18:2/23:0)** | **634.612** | **9.907** |
| **LMSP02010021** | **C41H81NO3** | **Cer(d18:1/23:0)** | **636.626** | **11.018** |
| **LMSP02010009** | **C42H81NO3** | **Cer(d18:1/24:1(15Z))** | **670.610** | **9.979** |
| **LMSP02050007** | **C42H82NO6P** | **CerP(d18:1/24:1(15Z))** | **728.597** | **3.513** |
| **LMSP02020012** | **C42H85NO3** | **Cer(d18:0/24:0)** | **674.639** | **11.850** |
| **LMSP02030004** | **C42H85NO4** | **Cer(t18:0/24:0)** | **690.635** | **11.601** |
| **LMSP02030002** | **C42H85NO5** | **Cer(t18:0/h24:0)** | **706.629** | **10.505** |
| **LMSP02010010** | **C44H85NO3** | **Cer(d18:1/26:1(17Z))** | **698.643** | **11.672** |
| **LMSP02010011** | **C44H87NO3** | **Cer(d18:1/26:0)** | **678.674** | **13.066** |
| **LMSP02020014** | **C44H89NO3** | **Cer(d18:0/26:0)** | **680.692** | **9.730** |
| **LMSP02030003** | **C44H89NO5** | **Cer(t18:0/h26:0)** | **712.678** | **9.161** |
| **LMSP02050011** | **C46H90NO6P** | **CerP(d18:1/26:1(17Z))** | **784.658** | **4.949** |
| **LMSP02040001** | **C66H125NO5** | **N-(30-(9Z,12Z-octadecadienoyloxy)-triacontanoyl)-sphing-4-enine** | **1012.959** | **16.517** |
